# Predictors of mortality from diarrhea during a cholera outbreak in Haiti’s Artibonite and Centre regions, 2022–2024

**DOI:** 10.1371/journal.pntd.0014626

**Published:** 2026-08-14

**Authors:** Yodeline Guillaume, Ralph Ternier, Jean J. Manasse, Marc A. Jeune, Alain Casseus, Wesler Lambert, Wilfredo R. Matias, Molly F. Franke, Louise C. Ivers

**Affiliations:** 1 Center for Global Health, Massachusetts General Hospital, Boston, Massachusetts, United States of America; 2 Zanmi Lasante, Croix-des-Bouquets, Haiti; 3 Division of Infectious Diseases, Brigham and Women’s Hospital, Boston, Massachusetts, United States of America; 4 Division of Infectious Diseases, Massachusetts General Hospital, Boston, Massachusetts, United States of America; 5 Division of Global Health Equity, Brigham and Women’s Hospital, Boston, Massachusetts, United States of America; 6 Department of Global Health and Social Medicine, Harvard Medical School, Boston, Massachusetts, United States of America; 7 Department of Epidemiology, Harvard T. H. Chan School of Public Health, Boston, Massachusetts, United States of America; 8 Harvard Global Health Institute, Cambridge, Massachusetts, United States of America; Mahidol Univ, Fac Trop Med, THAILAND

## Abstract

**Background:**

Haiti reported a resurgence of cholera in October 2022, which caused 88,087 suspected cases and 1,324 deaths through December 2024. There is limited data on the predictors of mortality from cholera during this epidemic period.

**Methodology/Principal Findings:**

We conducted a retrospective cohort analysis of data from 15 cholera treatment centers (CTCs) supported by a non-governmental health organization in Haiti to identify factors associated with suspected cholera deaths. Cases were individuals treated for acute watery diarrhea at CTCs in the Artibonite and Centre departments between October 2022 and December 2024. We described the characteristics of cases, calculated case fatality ratios (CFRs), and used mixed-effects logistic regression to assess predictors of death, accounting for clustering by CTC. Missing data were addressed using multiple imputation chained equations (MICE, m = 50). We analyzed 17,912 suspected cholera cases, including 134 deaths (overall CFR: 0.7%). Nearly 51.0% of cases were male (9,056/17,822), and 32.2% (5,688/17,659) were aged ≤5 years. A quarter (4,490/17,912) of all cases presented with severe dehydration, and 65.1% (8,951/13,759) reported vomiting. After accounting for facility-level clustering, mortality was significantly associated with age ≥ 60 years (adjusted odds ratio [aOR] 6.08, 95%CI 3.56 - 10.38, p < 0.001), severe dehydration (aOR 5.24, 95%CI 2.85 - 9.64, p < 0.001), and vomiting (aOR 2.96, 95%CI 1.33 - 6.56, p = 0.008). Odds of death were lower among cases treated in 2023 (aOR 0.32, 95%CI 0.20 - 0.50, p < 0.001) and 2024 (aOR 0.24, 95%CI 0.07 - 0.79, p = 0.02), compared with 2022.

**Conclusions/Significance:**

Older age, severe dehydration, and vomiting were significant predictors of cholera-related deaths in the Centre and Artibonite regions during Haiti’s 2022–2024 cholera epidemic. Improving data collection during outbreaks, including information on comorbidities, may enhance our understanding of these associations and inform interventions for further reducing cholera mortality.

## Author summary

Cholera is an acute diarrheal disease that claims tens of thousands of lives globally each year, yet data on the predictors of mortality are scarce in many affected countries. Haiti experienced a resurgence of cholera in October 2022 after three years without a confirmed case. We analyzed data from 17,912 patients who sought care at cholera treatment centers in the Artibonite and Centre regions of Haiti from October 2022 to December 2024 to understand who was most at risk of dying during this epidemic period. Overall, fewer than 1% of patients died, but the risk of death was much higher among older adults, those with severe dehydration, and those who reported vomiting. These findings are largely consistent with existing evidence on predictors of cholera mortality but also reveal serious gaps in routine surveillance data, including a lack of information on comorbidities that likely play a role in mortality. Further, we identified that, in addition to diarrhea, a large portion of patients had vomiting at presentation, and this symptom was a predictor of mortality. Strengthening data quality could improve our understanding of high-risk groups for mortality, and guide strategies to reduce deaths.

## Introduction

Cholera is a centuries-old acute watery diarrheal disease caused by the bacterium *Vibrio cholerae*. Globally, it still kills between 21,000 and 143,000 people annually [1], with mortality rates remaining persistently high in some endemic regions [2]. Death from cholera is mainly due to hypovolemic shock, which results from rapid, severe fluid loss, without timely and adequate treatment [3]. Despite being both preventable and treatable, it remains a significant public health threat with the potential for high morbidity and mortality, particularly in settings with limited access to clean water, sanitation, and healthcare [4]. In countries such as Haiti, these conditions – worsened by humanitarian crises (e.g., conflict, natural disasters) – have contributed to large, recurring outbreaks and substantial deaths [5].

Existing research demonstrates that certain population groups are at higher risk of severe disease or death from cholera than others, including undernourished children [6], fetuses or neonates of women infected during pregnancy [7–9], and individuals immunologically naïve to the disease [3]. Some studies have further identified comorbidities (e.g., HIV infection, hypertension), advanced age (e.g., ≥ 50 years), late presentation for clinical care, and male sex as contributing factors, but these findings have not been consistently observed across settings [10,11]. Consequently, there remain gaps in our understanding of additional high-risk groups and specific predictors of mortality.

Over the past 15 years, Haiti has experienced several waves of devastating cholera outbreaks. After an initial epidemic that began in 2010, no cases were reported from March 2019 until a resurgence in October 2022, which caused 88,087 suspected cases and 1,324 deaths through December 2024 [12]. Given that there is little data on the most recent cholera epidemic in Haiti, we aimed to (1) describe the characteristics of suspected cholera cases treated at 15 health facilities in Haiti’s Artibonite and Centre regions, (2) estimate the case fatality ratio (CFR) by demographic strata and clinical symptoms at presentation, and (3) explore the predictors of cholera-related mortality.

## Methods

### Ethics statement

We analyzed de-identified data from a previously collected case-based dataset that was created for surveillance of diarrhea trends to fulfill facility reporting requirements and that did not require patient consent. The parent data system was co-created as one component of an overarching collaborative program of cholera control between Zanmi Lasante and Mass General Hospital. Data use for this sub-project was governed by a data use agreement between the two entities and analysis of de-identified data was reviewed and exempted by the Mass General Brigham Institutional Review Board (Protocol # 2025P001104) in Boston, Massachusetts, United States.

### Study design, setting, and population

We analyzed cohort data from an electronic registry of acute watery diarrhea (AWD) cases treated between October 2022 and December 2024 at 15 diarrhea or cholera treatment centers (CTCs) supported by a Haitian non-governmental organization (NGO) in Haiti’s Artibonite and Centre regions. We have previously described the work of this NGO, Zanmi Lasante [13]. The Artibonite and Centre are two of ten administrative regions of the country, marked by inadequate WASH infrastructure and high levels of poverty, and reported the second and third highest number of suspected cholera cases in the country, respectively, during the study period [12]. Based on the last Demographic Health Survey conducted in 2017, only 55.8% and 53.5% of households in the Artibonite and Centre, respectively, had an improved water source either located on the premises or within a 30-minute round-trip collection time [14]. In addition, only 23% of households in each region had an improved sanitation facility not shared with other households, and less than 19% each had a handwashing station with soap and water available. National estimates at the time of the resurgence of cholera in Haiti in 2022 showed persisting gaps in WASH infrastructure and nearly 59.0% of the population living below the poverty line [15,16].

The study population consisted of all individuals who presented at the CTCs for care during the epidemic period of interest. To be admitted to a CTC, patients had to have AWD, defined as three or more loose or watery, non-bloody stools within a 24-hour period. In addition, the standard registry form provided by the Haitian Ministry of Health (MoH) included a question asking data clerks to denote a case as “meeting the MoH definition of suspected cholera” or not. Throughout the study period, the MoH defined a suspected cholera case as “any person presenting with acute, watery, profuse diarrhea, with or without vomiting, with or without dehydration” [12,17]. Because it was uncertain how the definition was applied by data clerks in denoting a subset of admitted AWD patients, we decided *a priori* to use the World Health Organization’s (WHO) most inclusive definition of suspected cholera in the context of a confirmed outbreak: any person with AWD [18]. Therefore, our case definition was all medically attended persons with AWD at the CTCs.

### Data source and management

As part of its disease surveillance and monitoring system, the Haitian MoH requires health institutions to complete a paper or an electronic registry of AWD cases to record the number of patients seen daily and generate national-level weekly reports. To enhance the NGO’s response to local outbreaks and aid with planning of clinical care and management, we co-created an electronic registry by digitizing information from the 15 diarrhea or cholera treatment centers’ (CTCs) paper registries into a centralized database. Data entry occurred daily (or almost daily) and enabled the NGO to rapidly examine diarrhea incidence, trends, and outcomes in its catchment areas.

The electronic registry data consisted of a combination of patient- and clinician-reported information from admission to discharge or death within the CTCs. Demographic and clinical variables included patient’s age, sex, place of residence (administrative units known as ‘commune’, ‘communal section’, ‘locality’), referral in from another CTC or a formal oral rehydration point, time from onset of symptoms to presentation at the CTC (defined as either <24 hours or ≥ 24 hours), intake of oral rehydration solution (ORS) prior to presentation, dehydration status at presentation (none or mild, moderate, severe), presence of vomiting (yes/no). Additional variables of relevance to the analysis were oral cholera vaccination status, date of admission and discharge, outcome (i.e., discharged home, transferred, or died), and results of cholera rapid diagnostic tests (RDTs) when available. Other laboratory results and information on comorbidities were not captured in the registry.

Our primary outcome of interest was institutional death, defined by the MoH as death of a suspected or confirmed cholera case, with no other known cause of death, that occurs after admission to a health facility [12]. We considered all cases who were discharged to their home or transferred to another health facility as survivors. The selection of predictors (e.g., age, sex, time to presentation, clinical symptoms) for analysis was based on commonly reported predictors of mortality in the cholera literature [11] and availability in the dataset. Except for age and dates of admission and discharge, all other variables were binary or categorical. For each predictor, we calculated CFR by dividing the number of deaths within a given category or level of the predictor by the total number of cases observed in that same category, multiplied by 100.

### Statistical analysis

Patient characteristics were summarized using medians and interquartile ranges (IQRs) for continuous variables (e.g., age) and frequencies and percentages for binary and categorical variables (e.g., sex, dehydration status). Since several predictors had missing data, ranging from <1% for sex to 24.4% for vomiting, we assessed the missingness mechanism using logistic regression to estimate the odds of missingness based on the observed covariates, and determined that data were plausibly missing at random [19]. We performed multiple imputation by chained equations (MICE), generating 50 imputed datasets to address missing data on age, sex, referral, ORS prior to presentation, time to presentation, and vomiting. To identify factors associated with death, we used the pooled multiply imputed data to fit a univariable logistic regression model for each relevant covariate, with a random intercept for health facility clustering. We collapsed three CTCs from the Centre into a single cluster and four Artibonite CTCs into another, resulting in a total of ten clusters, to reduce small groupings and improve numerical stability during imputation. The variance-components matrix was estimated via QR decomposition to aid convergence near parameter boundaries [20]. Variables with a P-value <0.20 were subsequently included in a mixed-effects multivariable logistic regression model.

To validate the estimated associations, we performed several sensitivity analyses, including missing indicator and complete case analyses for missing data and an analysis restricted to the subset of AWD cases meeting the Haitian MoH’s definition of suspected cholera. We considered predictors with a P-value <0.05 to be statistically significant. We used Stata version 17 (Stata Corp. LP) for all analyses.

Sample size was determined based on the available data and our inclusion criteria (e.g., case definition, study period of interest). While *a priori* sample size calculations were not conducted, given the exploratory nature of our analysis, we performed a post hoc power analysis using Power and Sample (PS) Calculations software (version 3.1.6). Assuming a Type I error probability (α = 0.05), and no clustering, we would have 80% power to detect differences of at least 0.36 or 1.8 for a low-prevalence exposure (11%) and at least 0.6 or 1.5 for a high-prevalence exposure (50%). Holding other factors constant, clustering would be expected to push detectable differences further from the null.

## Results

**Fig 1:**
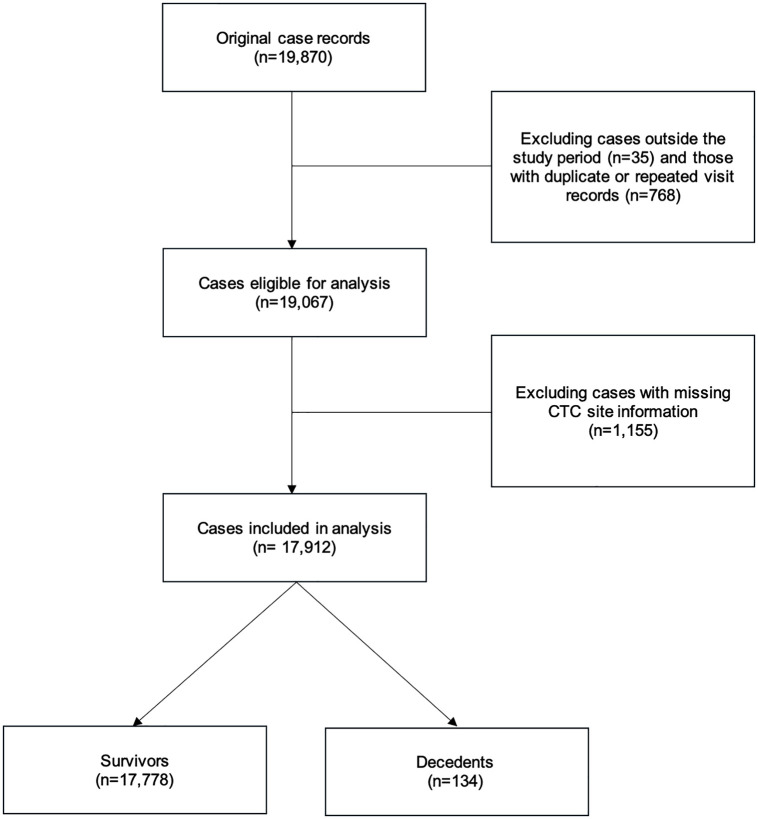
Selection of eligible cases for the full analysis of suspected cholera cases^a^ in 15 health facilities in the Artibonite and Centre, Haiti, during a confirmed cholera outbreak, 2022-2024. ^a^ Suspected cholera case defined by: Acute watery diarrhea and attendance for medical care at a cholera treatment center.

**Fig 2:**
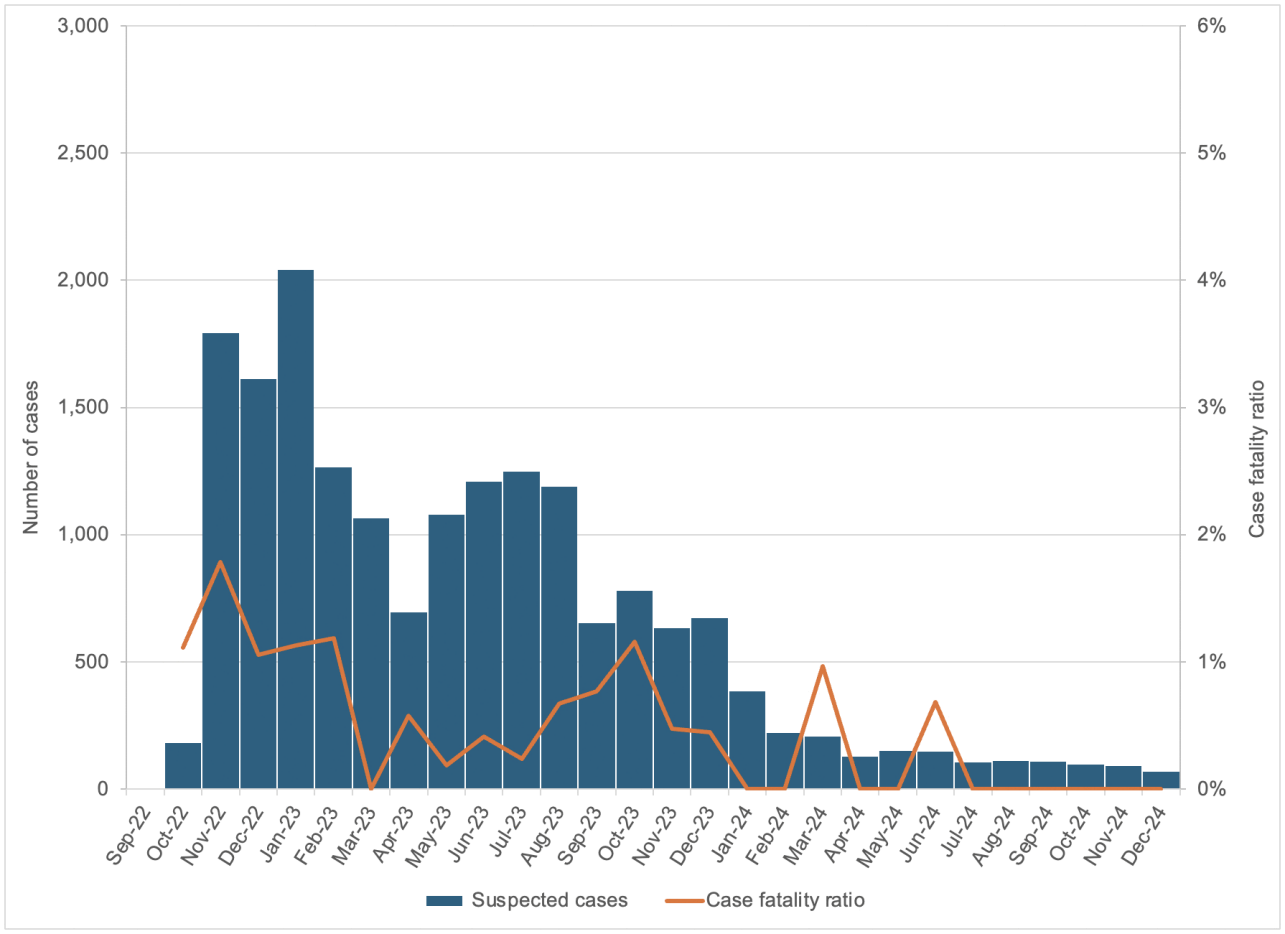
Distribution of 17,912 suspected cholera cases^a^ and case fatality ratios (CFR) reported by month in 15 health facilities in the Artibonite and Centre, Haiti, during a confirmed cholera outbreak, 2022-2024. ^a^ Suspected cholera case defined by: Acute watery diarrhea and attendance for medical care at a cholera treatment center.

Of 19,870 registry records, we excluded 1,958 (35 cases treated outside our study period, 768 duplicate and repeated visit records, and 1,155 with missing CTC site identifier) (see Fig 1 for the selection of eligible cases). We analyzed 17,912 cases, including 134 decedents, who were treated in 15 CTCs in the Artibonite and Centre regions between October 10, 2022 and December 28, 2024. Nearly 88% (15,225/17,400) of individuals were classified as meeting the MoH’s definition of a suspected cholera case. Fig 2 shows the distribution of cases and deaths over the two-year period, with three distinct waves: an initial peak between November 2022 and February 2023 immediately after the start of the new outbreak in October 2022, followed by some smaller waves in 2023 in the context of an overall declining trend.

The demographic and clinical characteristics of cases are summarized in Table 1, along with the associated CFRs. Overall, 76% of cases in the registry were treated in the Centre region. Nearly 51.0% (9,056/17,822) of cases were males. Children under five years old accounted for almost a third of all cases (5,688/17,659), the highest proportion of all age groups. Over half (9,551/15,283) of patients had symptoms lasting one day or more from symptom onset to presentation, but only 20.2% (3,577/17,690) took ORS prior to presentation. About 75.0% of all cases suffered from moderate or severe dehydration at presentation, and nearly two-thirds (8,951/13,759) reported vomiting. Less than three percent of individuals self-reported previously receiving an oral cholera vaccine (OCV), and there were no deaths among vaccinees. However, because the overall distribution was sparse and uneven across health facilities, with 92.9% of all vaccinees seen at just three CTCs, we did not include OCV receipt in additional analyses. A rapid diagnostic test for cholera was performed for only 4,964 individuals (27.7% of cases), primarily from four sites. Of those tested, 98.1% were positive for *V. cholerae* (See supplementary file, S1 Table).

**Table 1:** Characteristics of 17,912 suspected cholera cases^a^ in the Artibonite and Centre, Haiti, during a confirmed cholera outbreak, 2022-2024.

Variable	Overall	Survived	Deceased	CFR
	n (%)	n (%)	n (%)	%
	(n = 17,912)^b^	(n = 17,778) ^b^	(n = 134) ^b^	
Year of presentation				
2022 (Oct - Dec)	3,584 (20.0)	3,533 (19.9)	51 (38.1)	1.4
2023	12,519 (69.9)	12,439 (70.0)	80 (59.7)	0.6
2024	1,809 (10.0)	1,806 (10.2)	3 (2.2)	0.2
Location of CTC				
Artibonite	4,268 (23.8)	4,209 (23.7)	59 (44.0)	1.4
Centre	13,644 (76.2)	13,569 (76.3)	75 (56.0)	0.6
Age in years, median [IQR]	11 [3 - 32]	10.5 [3 - 32]	40 [11 - 68]	
Age group (N = 17,659)				
0-4	5,688 (32.2)	5,667 (32.2)	21 (16.0)	0.4
5-19	5,219 (29.6)	5,195 (29.6)	24 (18.3)	0.5
20-39	3,251 (18.4)	3,232 (18.4)	19 (14.5)	0.6
40-59	2,002 (11.3)	1,981 (11.3)	21 (16.0)	1.1
60+	1,499 (8.5)	1,453 (8.3)	46 (35.1)	3.1
Missing	253	250	3	1.2
Sex (N = 17,822)				
Male	9,056 (50.8)	8,979 (50.8)	77(58.8)	0.9
Female	8,766 (49.2)	8,712 (49.2)	54 (41.2)	0.6
Don’t know/Missing	90	87	3	3.3
Referred to treating health facility (N=15,016)				
No	14,581 (97.1)	14,483 (97.1)	98 (95.1)	0.7
Yes	435 (2.9)	430 (2.9)	5 (4.9)	1.2
Don’t know/Missing	2,896	2,865	31	1.1
Time from symptom onset to presentation (N = 15,283)				
Less than 24 hours	5,732 (37.5)	5,683 (37.4)	49 (45.8)	0.9
24 hours or more	9,551 (62.5)	9,493 (62.6)	58 (54.2)	0.6
Don’t know/Missing	2,629	2,602	27	1.0
Oral rehydration solution intake prior to presentation (N = 17,690)				
No	14,113 (79.8)	14,001 (79.7)	112 (87.5)	0.8
Yes	3,577 (20.2)	3,561 (20.3)	16 (12.5)	0.5
Don’t know/Missing	222	216	6	2.7
Vomiting (N = 13,759)				
No	4,808 (34.9)	4,801(35.2)	7 (6.6)	0.2
Yes	8,951 (65.1)	8,852 (64.8)	99 (93.4)	1.1
Don’t know/Missing	4,153	4,125	28	0.7
Dehydration at admission				
None or mild	4,490 (25.1)	4,477 (25.2)	13 (9.7)	0.3
Moderate	8,988 (50.2)	8,959 (50.4)	29 (21.6)	0.3
Severe	4,434 (24.8)	4,342 (24.4)	92 (68.7)	2.1
Self-reported oral cholera vaccination (N = 16,806)				
No	16,372 (97.4)	16,249 (97.4)	123 (100.0)	0.8
Yes	434 (2.6)	434 (2.6)	0 (0.0)	0.0
Don’t know/Missing	1,106	1,095	11	1.0

^a^Suspected cholera case defined by: Acute watery diarrhea and attendance for medical care at a cholera treatment center.

^b^Unless otherwise specified.

Cases treated at the CTCs in the Artibonite had a higher CFR than those in the Centre (1.4% vs. 0.6%). Older individuals had the highest age-specific CFRs, ranging from 1.1% among those aged 40–59 years to 3.1% among those ≥60 years. CFR was also high among cases with severe dehydration (2.1%) or vomiting (1.1%).

In univariable analysis assessing predictors of mortality and accounting for clustering at the facility-level, odds of death were significantly higher among those aged 40–59 years old (odds ratio [OR] 2.35, 95%CI 1.27 - 4.34, p = 0.007), and ≥ 60 years old (OR 7.25, 95%CI 4.28 - 12.28, p < 0.001), compared with children under 5 years old (see Table 2). Similarly, severe dehydration (OR 6.36, 95%CI 3.51 - 11.52, p < 0.001) and vomiting (OR 3.97, 95%CI 1.83 - 8.61, p = 0.001) were associated with greater odds of death in contrast to those with no or mild dehydration and no vomiting, respectively. Factors associated with significantly lower odds of death included presenting to CTCs in 2023 (OR 0.40, 95%CI 0.27 - 0.61, p < 0.001) or 2024 (OR 0.25, 95%CI 0.07 - 0.83, p = 0.02), compared with 2022.

**Table 2:** Predictors of death from suspected cholera^a^ in the Artibonite and Centre, Haiti, during a confirmed cholera outbreak, 2022-2024. (N = 17,912).

	Univariable analysis			Multivariable analysis		
	Unadjusted odds ratio (95% CI)	Standard error	P-value	Adjusted odds ratio (95% CI)	Standard error	P-value
Year of presentation						
2022 (Oct - Dec)	Reference			Reference		
2023	0.40 (0.27–0.61)	0.08	<0.001	0.32 (0.20–0.50)	0.07	<0.001
2024	0.25 (0.07–0.83)	0.15	0.02	0.24 (0.07–0.79)	0.15	0.02
Age group						
0-4	Reference			Reference		
5-19	1.09 (0.60–1.97)	0.33	0.78	0.88 (0.48–1.59)	0.27	0.67
20-39	1.31 (0.70–2.45)	0.42	0.40	1.08 (0.57–2.02)	0.34	0.82
40-59	2.35 (1.27–4.34)	0.74	0.007	1.82 (0.98–3.38)	0.58	0.06
60+	7.25 (4.28–12.28)	1.95	<0.001	6.08 (3.56–10.38)	1.66	<0.001
Sex						
Female	Reference			Reference		
Male	1.37 (0.97–1.94)	0.24	0.08	1.33 (0.93–1.90)	0.24	0.12
Referred to treating health facility						
No	Reference					
Yes	1.43 (0.57–3.56)	0.66	0.45			
Time from symptom onset to presentation						
Less than 24 hours	Reference			Reference		
24 hours or more	0.59 (0.37–0.94)	0.14	0.03	0.59 (0.37–0.94)	0.14	0.03
Oral rehydration solution intake prior to presentation						
No	Reference					
Yes	0.74 (0.43–1.28)	0.21	0.28			
Vomiting						
No	Reference			Reference		
Yes	3.97 (1.83–8.61)	1.56	0.001	2.96 (1.33–6.56)	1.20	0.008
Dehydration at admission						
None or mild	Reference			Reference		
Moderate	1.10 (0.57–2.14)	0.37	0.78	1.02 (0.52–2.01)	0.35	0.94
Severe	6.36 (3.51–11.52)	1.93	<0.001	5.24 (2.85–9.64)	1.63	<0.001

^a^Suspected cholera case defined by: Acute watery diarrhea and attendance for medical care at a cholera treatment center.

In the mixed-effects multivariable logistic analysis, after adjusting for CTC site, year of presentation, older age, severe dehydration, and vomiting remained significant predictors of death from AWD. Cases aged ≥ 60 years had six-fold odds of death (adjusted OR [aOR] 6.08, 95%CI 3.56 - 10.38, p < 0.001). Severe dehydration (aOR 5.24, 95%CI 2.85 - 9.64, p < 0.001) and vomiting (aOR 2.96, 95%CI 1.33 - 6.56, p = 0.008) were associated with significantly higher odds of death in this population, compared to those with no or mild dehydration and no vomiting, respectively. By contrast, presenting 24 hours or more after symptom onset was inversely associated with death (aOR 0.59, 95% CI 0.37 - 0.94, p = 0.03).

We evaluated multicollinearity among the predictors (including vomiting and severe dehydration) using the variance inflation factor (VIF). VIF values were low across all imputed datasets, with a maximum of 1.61 for severe dehydration. The average residual intraclass correlation coefficient across the imputed datasets was 0.18, suggesting meaningful clustering by facility (i.e., cases within the same CTC had more similar odds of deaths than patients from different facilities). Results were generally consistent in sensitivity analyses restricting the analysis to the complete case data or to only the subset of AWD cases that met the Haitian MoH’s definition of suspected cholera case (See S2-S4 Tables) and using a missing-indicator method to manage missing data in lieu of the MICE approach (S5 Table).

## Discussion

This study on the clinical characteristics and predictors of mortality in AWD patients medically attended at 15 CTCs during a cholera epidemic in Haiti from 2022 to 2024 has some key strengths. It includes a large cohort of cases, uses data from 15 CTCs across two major regions (Artibonite and Centre) of the country, and covers multiple years. It also reflects real programmatic conditions, while reporting a case-fatality ratio below 1%, which is an important public health indicator for quality of care [21].

The overall observed CFR of 0.7% is similar to the CFRs for the study regions (each 0.8%) and nationwide (1.2%) based on the Haitian MoH situation reports over the same period [12]. However, for the first three months of the epidemic, our observed CFR (1.4%) is lower than the nationally reported 14-day CFR (3.0%) [22]. Although death from diarrhea is rare when adequate interventions and treatment are provided, progress on global case fatality rates has generally now stalled, and, in some outbreaks, mortality has increased [4]. CFR in our cohort is considered ‘acceptable’ on aggregate based on general guidance of a minimum CFR threshold of <1% in treatment facilities [21,23] and is consistent with our prior reports from the 2013–2017 epidemic period in Haiti [13]. This illustrates that case fatality ratios below the ‘acceptable’ standard are indeed achievable even in health systems with limited resources. Most deaths from cholera should be preventable with appropriate staff, systems, supplies, and support. We have previously described Zanmi Lasante’s approach to early identification, training, and case management of cholera within a comprehensive multi-sectoral public health response including water, sanitation, hygiene and OCV [13].

We observed that CFR decreased over the three calendar years of this outbreak – a phenomenon that is often documented in outbreak settings. The reasons for this require further study as current routine data collection in Haiti – and most humanitarian settings – does not permit a fuller understanding of causal factors, either system- or patient-related. The improvement in CFR could, for example, reflect a gradual strengthening of the outbreak response over time. Since Haiti had not reported a confirmed cholera case for the three years prior to the resurgence in 2022, health facilities may have required some time to establish or re-establish human resources and supply chain to respond to and provide care to a large volume of patients with diarrhea. There may have also been shifts in patient-related health-seeking factors, but these have not been fully studied. One would expect that with increased public health messaging and community awareness, patients would progressively seek care earlier in the course of their illness. In our analysis, time from symptom onset to presentation was inversely associated with death. A similar finding has been reported for Mozambique [24], and could indicate a survivor bias, whereby more severe cases die before reaching a health facility while those presenting later may have milder illness.

Overall, there was a higher burden of cases among children under 5 and those aged 5–19 years compared to older adults, which is consistent with the epidemiology of cholera in other countries, as limited prior natural exposure increases susceptibility [25]. However, older adults were more likely to die than their younger counterparts in our study, with age ≥ 60 years old being a significant predictor of death. Previous studies of patients with medically-attended cholera in Haiti during the 2010–2011 epidemic and in several African countries including Congo, Guinea-Bissau, Nigeria, and Zambia have reported a similar association, and suggest that immunosenescence and pre-existing comorbidities may complicate cholera treatment in older patients [26–29]. We were, however, unable to assess the underlying prevalence of comorbidities including malnutrition in children, or hypertension for example, as such data are not routinely documented in the national registry, and medical records in this humanitarian setting were sparse.

A better understanding of the association between age and cholera mortality is important for improved triaging and may offer pathways to reduce mortality: individualized care for adults with suspected cholera, including attention to their comorbidities, is not typically included in cholera case management protocols [30]. For example, the prevalence of hypertension in Haiti among adults over 40 years of age is estimated to be 24–70% [31,32] and of heart failure to be 3.8% (higher than in high-income countries) [33]. Both of these diseases may complicate the management of severe dehydration, and comorbidities such as diabetes and HIV have been found to be important predictors of cholera mortality in other contexts [10]. Routine collection of data on existing comorbidities in AWD registries during cholera outbreaks may therefore be important for better identifying groups at higher risk of death.

Our study showed a consistent association between severe dehydration on presentation and risk of death, which is unsurprising as hypovolemic shock is the classic cause of death in cholera, and clinical assessment for and management of dehydration the core of case management. In line with our findings, studies of cholera in Nigeria and South Sudan have reported a four- to five-fold increased odds of death with severe dehydration [34,35]. In addition, vomiting in our cohort was associated with increased risk of mortality, and patients presenting with vomiting had nearly three-fold odds of dying. Vomiting has previously been linked with fetal death among pregnant women with cholera in Haiti [7,8], but not separately from dehydration in the general patient population, as the low collinearity between these variables suggests in our study. While vomiting may be a marker of severe cholera illness [35], it could also be a proxy for being unable to tolerate ORS, or for another pathophysiological issue. For example, patients with vomiting may have more severe electrolyte derangements, or kidney injury.

We further found that, after accounting for the patient-level factors, 18% of the variation in log-odds of death was attributable to differences between CTCs (as indicated by a residual intraclass correlation of 0.18). This may be due to clustering of comorbidities or other markers of increased mortality rate for which we could not account. Additionally, facility-level factors such as quality of care, case management protocols, and staffing can impact cholera outcomes. For example, being treated in a health facility with less than 0.5 nurses per two patients have been associated with higher odds of cholera-related death in Cameroon [36], while being treated in government-operated health facilities in Kenya lowered risk of mortality [37]. Our study did not encompass a health-facility level assessment of the CTCs to further evaluate the likely factors behind this variance.

Less than half of all cases sought medical care for their diarrheal episode within 24 hours of symptom onset, and only 20% took home-based ORS before presenting to a CTC. Although these factors were not significantly associated with mortality in the multivariable analysis, they are noteworthy observations in a country that already had nearly 10 years of experience with the management and prevention of cholera prior to the resurgence in 2022 and signify important opportunities for community management. Our research over the past decade in these regions consistently shows high levels of knowledge about cholera among residents of these areas, and low access to the preventive and treatment modalities in the community [38]. A protracted sociopolitical crisis in Haiti since 2018 may have further restricted access to basic resources and increased vulnerability to the effects of cholera [39].

Our study had limitations. We included multiple CTCs, but they may not be representative of other CTCs in the two regions or across Haiti. However, the cohort is the largest analysis of predictors of mortality from the country, and the included regions are generally underserved, lacking in many basic infrastructural supports, with a high prevalence of extreme poverty and, in those ways, resemble many regions of Haiti and other cholera-afflicted regions of the world. Institutional deaths are only one component of the impact of cholera outbreaks, and the surveillance registry we analyzed does not permit estimation of community deaths, which in previous studies have been high [40,41]. By analyzing individuals who presented to a CTC, we may have systematically excluded people who died quickly before seeking care, biasing the estimate for time from symptom onset to presentation.

We used the WHO’s broad case definition of suspected cholera, considering all medically attended AWD during the outbreak as suspected cholera [18]. However, because the signs and symptoms of mild to moderate cholera can overlap with those of other diarrheal diseases, AWD caused by other enteropathogens may have been attributed to cholera, leading to misclassification and an overestimation of cholera cases [42]. Rapid diagnostic testing for *V. cholerae* was performed without any particular regularity or protocol, and ultimately for about a quarter of cases. Therefore, we did not use RDT positivity as a primary inclusion criterion for our analysis. Yet, more than 95% of those tested were in fact positive for *V. cholerae*, albeit other studies have reported lower RDT positivity [43]. A sensitivity analysis using the Haitian MoH’s more limited definition of suspected cholera case did not change our findings, including the aggregate case fatality ratio (see S4 Table). Lastly, some key variables had a large proportion of missing data, including OCV receipt, which was excluded from the analysis partly due to low self-reported vaccination across CTCs. Although a large OCV campaign took place in 2022, and smaller ones in 2023 and 2024 [17,44], our study was not designed to evaluate the effectiveness of OCV on CFR. Nevertheless, our results remained robust across the selected strategies for handling missingness.

## Conclusion

This study analyzes a cohort of 17,912 patients with medically-attended acute watery diarrhea in two regions of Haiti during the 2022–2024 cholera epidemic. The overall case fatality ratio was 0.7% but varied by epidemic year. Older age, severe dehydration, and vomiting emerged as significant predictors of mortality. Our findings add to the evidence on predictors of cholera mortality in the Haitian context during a resurgence of cholera while highlighting serious data gaps that limit our ability to identify intervenable variables for improved case management and outbreak response. Although data collection is challenging in fragile health systems and during humanitarian crises, we recommend strengthening the quality of surveillance data systems to help advance our understanding of the associations between patient characteristics and poor outcomes, and to inform strategies for further reducing cholera-related deaths.

## Supporting information

S1 Table Results of cholera rapid diagnostic test during a cholera outbreak in the Artibonite and Centre, Haiti, 2022–2024. (N = 4,964).
(DOCX)

S2 Table Characteristics of 10,385 suspected cholera cases^a^ during an outbreak in the Artibonite and Centre, Haiti, 2022–2024. (DOCX)

S3 Table Predictors of death from suspected cholera^a^ during an outbreak in the Artibonite and Centre, Haiti, 2022–2024. (N = 10,385).
(DOCX)

S4 Table Predictors of death from suspected cholera^a^ during an outbreak in the Artibonite and Centre, Haiti, 2022–2024. (N = 15,225).
(DOCX)

S5 Table Predictors of death from suspected cholera^a^ during an outbreak in the Artibonite and Centre, Haiti, 2022–2024. (N = 17,912).
(DOCX)
