# Supplementary material for: Predictors of mortality from diarrhea during a cholera outbreak in Haiti’s Artibonite and Centre regions, 2022–2024
Source: PLoS Negl Trop Dis. 2026 Aug 14;20(8):e0014626. doi: 10.1371/journal.pntd.0014626 (PMC13475942; doi:10.1371/journal.pntd.0014626)
Supplement: Supplementary file 1 [file pntd.0014626.s001.docx]

A. **RESULTS OF CHOLERA RAPID DIAGNOSIS TEST**

**S1 Table. Results of cholera rapid diagnostic test during a cholera outbreak in the Artibonite and Centre, Haiti, 2022-2024. (N=4,964)**

| **Cholera rapid diagnostic test result** | **Overall** | **Survived** | **Died** | **CFR** |
| --- | --- | --- | --- | --- |
|  | n (%) | n (%) | n (%) | % |
|  | (n=4,964) | (n=4,949) | (n=15) |  |
| Positive | 4,871 (98.1) | 4,857 (98.1) | 14 (93.3) | 0.3 |
| Negative | 93 (1.9) | 92 (1.9) | 1 (6.7) | 1.1 |
