# Supplementary material for: Predictors of mortality from diarrhea during a cholera outbreak in Haiti’s Artibonite and Centre regions, 2022–2024
Source: PLoS Negl Trop Dis. 2026 Aug 14;20(8):e0014626. doi: 10.1371/journal.pntd.0014626 (PMC13475942; doi:10.1371/journal.pntd.0014626)
Supplement: Supplementary file 2 [file pntd.0014626.s002.docx]

**B. COMPLETE CASE ANALYSIS** (except for self-reported oral cholera vaccination and the available rapid diagnostic test results, which are excluded from the mixed-effects logistic regression analysis)

**S2 Table. Characteristics of 10,385 suspected cholera cases^a^ during an outbreak in the Artibonite and Centre, Haiti, 2022-2024.**

| **Variable** | **Overall** | **Survived** | **Died** | **CFR** |
| --- | --- | --- | --- | --- |
|  | n (%) | n (%) | n (%) | % |
|  | (n=10,385) | (n=10,385) | (n=10,385) |  |
| **Year of presentation** |  |  |  |  |
| 2022 (Oct - Dec) | 1,309 (12.6) | 1,289 (12.5) | 20 (25.0) | 1.5 |
| 2023 | 7,695 (74.1) | 7,638 (74.1) | 57 (71.2) | 0.7 |
| 2024 | 1,381 (13.3) | 1,378 (13.4) | 3 (3.8) | 0.2 |
| **CTC location** |  |  |  |  |
| Artibonite | 2,305 (22.2) | 2,269 (22.0) | 36 (45.0) | 1.6 |
| Centre | 8,080 (77.8) | 8,036 (78.0) | 44 (55.0) | 0.5 |
| **Age in years,** median, [IQR] | 12 [3, 34] | 12 [3, 34] | 43.5 [13.5, 70] |  |
| **Age group** (in years) |  |  |  |  |
| 0-4 | 3,234 (31.1) | 3,220 (31.2) | 14 (17.5) | 0.4 |
| 15-9 | 3,008 (29.0) | 2,996 (29.1) | 12 (15.0) | 0.4 |
| 20-39 | 1,937 (18.7) | 1,926 (18.7) | 11 (13.8) | 0.6 |
| 40-59 | 1,247 (12.0) | 1,235 (12.0) | 12 (15.0) | 1.0 |
| 60+ | 959 (9.2) | 928 (9.0) | 31 (38.8) | 3.2 |
| **Sex** |  |  |  |  |
| Female | 5,162 (49.7) | 5,134 (49.8) | 28 (35.0) | 0.5 |
| Male | 5,223 (50.3) | 5,171 (50.2) | 52 (65.0) | 1.0 |
| **Referred to treating health facility** |  |  |  |  |
| No | 10,107 (97.3) | 10,030 (97.3) | 77 (96.2) | 0.8 |
| Yes | 278 (2.7) | 275 (2.7) | 3 (3.8) | 1.1 |
| **Time from symptom onset to presentation** |  |  |  |  |
| Less than 24 hours | 4,008 (38.6) | 3,973 (38.6) | 35 (43.8) | 0.9 |
| 24 hours or more | 6,377 (61.4) | 6,332 (61.4) | 45 (56.2) | 0.7 |
| **Oral rehydration solution intake prior to presentation** |  |  |  |  |
| No | 8,112 (78.1) | 8,038 (78.0) | 74 (92.5) | 0.9 |
| Yes | 2,273 (21.9) | 2,267 (22.0) | 6 (7.5) | 0.3 |
| **Vomiting** |  |  |  |  |
| No | 4,071 (39.2) | 4,066 (39.5) | 5 (6.2) | 0.1 |
| Yes | 6,314 (60.8) | 6,239 (60.5) | 75 (93.8) | 1.2 |
| **Dehydration at admission** |  |  |  |  |
| None or mild | 2,738 (26.4) | 2,733 (26.5) | 5 (6.2) | 0.2 |
| Moderate | 5,221 (50.3) | 5,199 (50.5) | 22 (27.5) | 0.4 |
| Severe | 2,426 (23.4) | 2,373 (23.0) | 53 (66.2) | 2.2 |
| **Self-reported oral cholera vaccination** |  |  |  |  |
| No | 10,084 (98.5) | 10,005 (98.5) | 79 (100.0) | 0.8 |
| Yes | 154 (1.5) | 154 (1.5) | 0 (0.0) | 0.0 |
| Missing | 147 | 146 | 1 | 0.7 |
| **Cholera rapid diagnostic test results** |  |  |  |  |
| Positive | 4,015 (98.0) | 4,005 (98.0) | 10 (100.0) | 0.3 |
| Negative | 82 (2.0) | 82 (2.0) | 0 (0.0) | 0.0 |
| Missing | 6,288 | 6,218 | 70 | 1.1 |

^a^ Suspected cholera case defined by: Acute watery diarrhea and attendance for medical care at a cholera treatment center
