# Supplementary material for: Predictors of mortality from diarrhea during a cholera outbreak in Haiti’s Artibonite and Centre regions, 2022–2024
Source: PLoS Negl Trop Dis. 2026 Aug 14;20(8):e0014626. doi: 10.1371/journal.pntd.0014626 (PMC13475942; doi:10.1371/journal.pntd.0014626)
Supplement: Supplementary file 3 [file pntd.0014626.s003.docx]

**B. COMPLETE CASE ANALYSIS**

**S3** **Table. Predictors of death from suspected cholera^a^ during an outbreak in the Artibonite and Centre, Haiti, 2022-2024. (N=10,385)**

|  | **Univariable model**^b^ | | | **Multivariable model**^b,c^ | | |
| --- | --- | --- | --- | --- | --- | --- |
|  | **Unadjusted odds ratio (95% CI)** | **Standard error** | **P-value** | **Adjusted odds ratio (95% CI)** | **Standard error** | **P-value** |
| **Year of presentation** |  |  |  |  |  |  |
| 2022 (Oct - Dec) | Reference |  |  | Reference |  |  |
| 2023 | 0.25 (0.13 - 0.48) | 0.08 | <0.001 | 0.24 (0.11 - 0.54) | 0.10 | <0.001 |
| 2024 | 0.29 (0.08 - 1.04) | 0.19 | 0.06 | 0.24 (0.06 - 0.87) | 0.16 | 0.03 |
| **Age group** |  |  |  |  |  |  |
| 0-4 | Reference |  |  | Reference |  |  |
| 5-19 | 0.76 (0.35 - 1.65) | 0.30 | 0.48 | 0.63 (0.29 - 1.38) | 0.25 | 0.25 |
| 20-39 | 1.01 (0.46 - 2.26) | 0.41 | 0.97 | 0.85 (0.38 - 1.89) | 0.35 | 0.68 |
| 40-59 | 1.74 (0.79 - 3.80) | 0.69 | 0.17 | 1.45 (0.66 - 3.19) | 0.58 | 0.36 |
| 60+ | 6.3 (3.30 - 12.01) | 2.07 | <0.001 | 5.52 (2.86 - 10.65) | 1.85 | <0.001 |
| **Sex** |  |  |  |  |  |  |
| Female | Reference |  |  | Reference |  |  |
| Male | 1.80 (1.14 - 2.86) | 0.43 | 0.013 | 1.79 (1.12 - 2.88) | 0.43 | 0.02 |
| **Referred to treating health facility** |  |  |  |  |  |  |
| No | Reference |  |  |  |  |  |
| Yes | 1.13 (0.35 - 3.66) | 0.68 | 0.83 |  |  |  |
| **Time from symptom onset to presentation** |  |  |  |  |  |  |
| Less than 24 hours | Reference |  |  |  |  |  |
| 24 hours or more | 0.75 (0.43 - 1.31) | 0.21 | 0.31 |  |  |  |
| **Oral rehydration solution intake prior to presentation** |  |  |  |  |  |  |
| No | Reference |  |  | Reference |  |  |
| Yes | 0.49 (0.21 - 1.15) | 0.21 | 0.10 | 0.59 (0.25 - 1.41) | 0.26 | 0.24 |
| **Vomiting** |  |  |  |  |  |  |
| No | Reference |  |  | Reference |  |  |
| Yes | 5.78 (2.24 - 14.88) | 2.79 | <0.001 | 4.52 (1.73 - 11.84) | 2.22 | 0.002 |
| **Dehydration at admission** |  |  |  |  |  |  |
| None or mild | Reference |  |  | Reference |  |  |
| Moderate | 1.93 (0.72 - 5.17) | 0.97 | 0.19 | 1.53 (0.56 - 4.16) | 0.78 | 0.40 |
| Severe | 8.24 (3.18 - 21.34) | 4.00 | <0.001 | 5.47 (2.09 - 14.34) | 2.69 | 0.001 |

^a^Suspected cholera case defined by: Acute watery diarrhea and attendance for medical care at a cholera treatment center**.**

^b^Mixed-effects logistic regression model using the complete case data of 10,385 patients (including 80 deaths) and a random intercept for health facility clustering. Five of 14 sites in the complete case data were combined into one cluster due to small number of cases, resulting in a total of 9 clusters in the analysis. The combined sites were all from the Centre region and included between 1 and 22 patients each.

^c^Residual intraclass correlation coefficient for the multivariable model: 0.15.
