# Supplementary material for: Predictors of mortality from diarrhea during a cholera outbreak in Haiti’s Artibonite and Centre regions, 2022–2024
Source: PLoS Negl Trop Dis. 2026 Aug 14;20(8):e0014626. doi: 10.1371/journal.pntd.0014626 (PMC13475942; doi:10.1371/journal.pntd.0014626)
Supplement: Supplementary file 4 [file pntd.0014626.s004.docx]

**C. ANALYSIS ON MULTIPLY IMPUTED DATA RESTRICTED TO PATIENTS WHO MET THE MINISTRY OF HEALTH’S DEFINITION OF SUSPECTED CHOLERA CASE**

**S4 Table. Predictors of death from suspected cholera**^a^ **during an outbreak in the Artibonite and Centre, Haiti, 2022-2024.** **(N=15,225)**

|  | **Multivariable model** ^b,c^ | | |
| --- | --- | --- | --- |
|  | **Adjusted odds ratio  (95% CI)** | **Standard error** | **P-value** |
| **Year of presentation** |  |  |  |
| 2022 (Oct - Dec) | Reference |  |  |
| 2023 | 0.35 (0.20 - 0.58) | 0.09 | <0.001 |
| 2024 | 0.22 (0.05 - 0.95) | 0.16 | 0.04 |
| **Age group** |  |  |  |
| 0-4 | Reference |  |  |
| 5-19 | 1.23 (0.61 - 2.47) | 0.44 | 0.57 |
| 20-39 | 1.20 (0.55 - 2.60) | 0.47 | 0.65 |
| 40-59 | 2.49 (1.20 - 5.14) | 0.92 | 0.014 |
| 60+ | 8.06 (4.24 - 15.32) | 2.64 | <0.001 |
| **Sex** |  |  |  |
| Female | Reference |  |  |
| Male | 1.30 (0.87 - 1.94) | 0.27 | 0.20 |
| **Time from symptom onset to presentation** |  |  |  |
| Less than 24 hours | Reference |  |  |
| 24 hours or more | 0.76 (0.46 - 1.24) | 0.19 | 0.27 |
| **Vomiting** |  |  |  |
| No | Reference |  |  |
| Yes | 3.43 (1.36 - 8.61) | 1.61 | 0.01 |
| **Dehydration at admission** |  |  |  |
| None or mild | Reference |  |  |
| Moderate | 1.76 (0.66 - 4.68) | 0.88 | 0.26 |
| Severe | 9.30 (3.67 - 23.56) | 4.41 | <0.001 |

^a^Denoted by data clerk as ‘meeting the Haitian Ministry of Health’s case definition,’ i.e., acute, watery, profuse diarrhea, with or without vomiting, with or without dehydration.

^b^Mixed-effect logistic regression on the multiply imputed data: Multivariable model restricted to 15,225 cases (including 106 deaths) who met the MoH’s case definition and using a random intercept for health facility clustering, an augmented estimation option to manage perfect prediction, and including all predictors from the univariable analysis with p<0.20.

^c^Residual intraclass correlation coefficient: 0.17.
