# Supplementary material for: Predictors of mortality from diarrhea during a cholera outbreak in Haiti’s Artibonite and Centre regions, 2022–2024
Source: PLoS Negl Trop Dis. 2026 Aug 14;20(8):e0014626. doi: 10.1371/journal.pntd.0014626 (PMC13475942; doi:10.1371/journal.pntd.0014626)
Supplement: Supplementary file 5 [file pntd.0014626.s005.docx]

**D. MISSING-INDICATOR ANALYSIS INCLUDING ALL CASES IN THE STUDY’S PRIMARY ANALYSIS (for comparison with the multiple imputation chained equations method for handling missingness)**

**S5 Table. Predictors of death from suspected cholera^a^ during an outbreak in the Artibonite and Centre, Haiti, 2022-2024. (N=17,912)**

|  | Multivariable model^b,c^ | | |
| --- | --- | --- | --- |
|  | **Adjusted odds ratio**  **(95% CI)** | **Standard**  **error** | **P-value** |
| Year of presentation |  |  |  |
| 2022 (Oct - Dec) | Reference |  |  |
| 2023 | 0.32 (0.20 - 0.52) | 0.08 | <0.001 |
| 2024 | 0.27 (0.08 - 0.91) | 0.17 | 0.03 |
| Age group |  |  |  |
| 0-4 | Reference |  |  |
| 5-19 | 0.89 (0.49 - 1.62) | 0.27 | 0.71 |
| 20-39 | 1.08 (0.57 - 2.02) | 0.35 | 0.82 |
| 40-59 | 1.88 (1.01 - 3.48) | 0.59 | 0.05 |
| 60+ | 6.41 (3.75 - 10.94) | 1.75 | <0.001 |
| Sex |  |  |  |
| Female | Reference |  |  |
| Male | 1.32 (0.93 - 1.89) | 0.24 | 0.13 |
| Time from symptom onset  to presentation |  |  |  |
| Less than 24 hours | Reference |  |  |
| 24 hours or more | 0.57 (0.36 - 0.89) | 0.13 | 0.02 |
| Vomiting |  |  |  |
| No | Reference |  |  |
| Yes | 3.47 (1.54 - 7.85) | 1.45 | 0.003 |
| Dehydration at admission |  |  |  |
| None or mild | Reference |  |  |
| Moderate | 1.03 (0.54 - 2.05) | 0.35 | 0.92 |
| Severe | 5.58 (3.04 - 10.27) | 1.74 | <0.001 |

^a^Suspected cholera case defined by: Acute watery diarrhea and attendance for medical care at a cholera treatment center.

^b^Mixed-effects logistic regression using missing indicators to handle missingness: We assigned the same fixed value of “99” to all missing observations and added indicator or dummy variables (with “1” for missing and “0” for not missing) in the multivariable model to indicate whether the value for a variable was missing. Model includes 17,912 cases (including 134 deaths) and all predictors from the univariable analysis with p<0.20.

^c^Residual intraclass correlation coefficient: 0.20.
